# Cytoplasmic Linker Protein-Associating Protein at the Nexus of Hormone Signaling, Microtubule Organization, and the Transition From Division to Differentiation in Primary Roots

**DOI:** 10.3389/fpls.2022.883363

**Published:** 2022-04-28

**Authors:** Laryssa Sophia Halat, Breanne Bali, Geoffrey Wasteneys

**Affiliations:** Department of Botany, University of British Columbia, Vancouver, BC, Canada

**Keywords:** root apical meristem, brassinosteroid (BR) signaling, auxin, PIN2, *Arabidopis thaliana*, sorting nexin 1 (SNX1), CLIP-associating protein (CLASP), cytokinin (CK)

## Abstract

The transition from cell division to differentiation in primary roots is dependent on precise gradients of phytohormones, including auxin, cytokinins and brassinosteroids. The reorganization of microtubules also plays a key role in determining whether a cell will enter another round of mitosis or begin to rapidly elongate as the first step in terminal differentiation. In the last few years, progress has been made to establish connections between signaling pathways at distinct locations within the root. This review focuses on the different factors that influence whether a root cell remains in the division zone or transitions to elongation and differentiation using *Arabidopsis thaliana* as a model system. We highlight the role of the microtubule-associated protein CLASP as an intermediary between sustaining hormone signaling and controlling microtubule organization. We discuss new, innovative tools and methods, such as hormone sensors and computer modeling, that are allowing researchers to more accurately visualize the belowground growth dynamics of plants.

## Introduction

Developmental transitions are a fact of life. All organisms, whether unicellular prokaryotes or multicellular eukaryotes, undergo transitions in response to endogenous or environmental cues. Doing this well ensures survival, and evolutionary success. Profound developmental switches are often associated with environmental stress and resource limitations. When deprived of key nutrients, microbes, including bacteria (Boutte and Crosson, 2013) and unicellular algae (Goodenough et al., 1995) enter dormancy until favorable conditions return. For land plants, local resource allocation is closely coupled with developmental programming to ensure that competing demands of different organs do not result in calamity. Plants use cues from the environment such as light, temperature, water and nutrients to determine when it is best to germinate, change growth direction, flower, release seeds, shed leaves or replace damaged or consumed organs.

Among the many remarkable things land plants do, coordinating what goes on above and below ground is perhaps the most important. During germination, the first priority is to get the cotyledons into the sunlight, so the finite resources housed in the seed are directed to elongating the hypocotyl at the expense of the root. But once the cotyledons unfurl, the resource allocation is reversed: hypocotyl expansion shuts down, and rapid root growth begins. Under drought conditions, shoot growth is curtailed, not only to conserve water, but also to direct photosynthesis-derived sucrose to the roots so that they can grow deeper to seek moisture. Investigating how root growth and development is modulated in response to prevailing environmental conditions – both below and above ground ones – has enormous potential for advancing the concept of resource allocation switching in organisms.

In this review, we cover recent advances in understanding the mechanisms that coordinate the transition from cell division to elongation in root apical meristems. We highlight recent findings that have brought to light a negative feedback-based regulatory mechanism involving three plant hormones, auxin, cytokinin and brassinosteroid (BR), and the microtubule-associated Cytoplasmic Linker Protein-Associating Protein (CLASP). We propose that this regulatory system, which involves two very distinct functions of CLASP, has evolved in land plants to modulate root apical meristem (RAM) activity to match development with prevailing environmental conditions.

## Root Growth: An Excellent Model for Understanding Developmental Transitions in Plants

**FIGURE 1:**
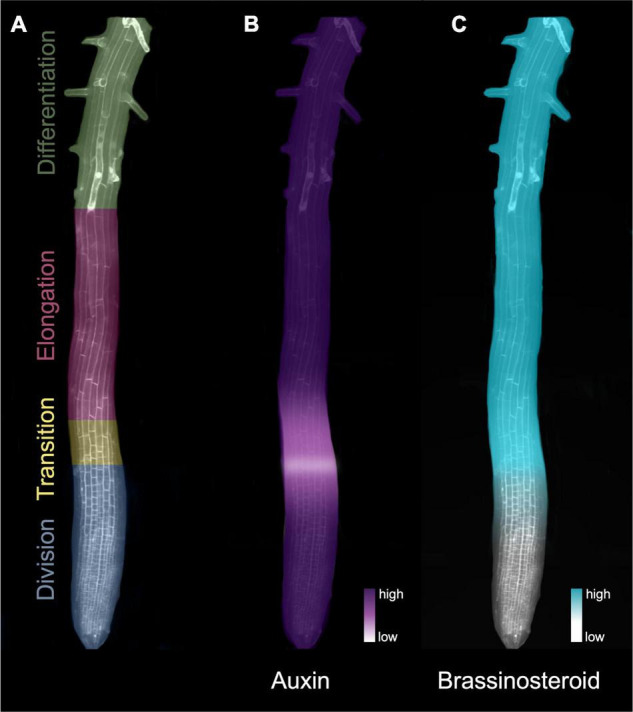
Hormone gradients define the developmental zones of the *Arabidopsis* root apical meristem. **(A)** Developmental zones of the root indicated in different colors. **(B)** The auxin signaling gradient in the root meristem. Auxin levels drop substantially at the beginning of the transition zone, forming an auxin minimum before increasing again toward the elongation zone. **(C)** The BR signaling gradient increases at the transition zone in the root meristem. The heatmaps in B) and C) denote levels of hormone (auxin) and signaling (BR).

*Arabidopsis thaliana* (hereafter *Arabidopsis*), the most widely used model organism for studying the molecular mechanisms and signaling pathways that govern root meristems (Sarkar et al., 2007), benefits from relatively simple architecture that is amenable to studying the developmental trajectory of different cell types. Its primary root has distinct developmental zones that can be tracked within each concentrically arranged tissue layer (Figure 1A). At the tip of the RAM, a group of cells termed the quiescent center is kept in a mitotically inactive state by the transcription factor WUSCHEL-RELATED HOMEOBOX5 (WOX5) (Forzani et al., 2014). In turn, the quiescent center cells transmit signals that prevent differentiation in the surrounding initials, thus maintaining stem cell identity (Van Den Berg et al., 1997). Formative cell divisions from the initial cells give rise to the distinct tissue layers followed by generative divisions that populate the division zones of the root tip, including the root cap below and beside the quiescent center, and the root proper, situated above it. The division zone contains cells that rapidly divide either anticlinally to produce more cells in the longitudinal direction, or occasionally laterally to generate more cell files.

Although some growth takes place in the division zone, it massively increases when cells cease dividing. This transition is marked by the development of large central vacuoles (Verbelen et al., 2006), and rapid, unidirectional expansion that is associated with mechanochemical modifications to the cell wall that increase extensibility (Vissenberg et al., 2000), while maintaining anisotropy through the synthesis of cellulose microfibrils (Wasteneys, 2004). Finally, root cells reach the differentiation zone, which is marked by cessation of cell elongation, and acquisition of inextensible secondary cell walls and other features. For the epidermal tissue, this involves the growth of protuberances or root hairs from cells in the trichoblast cell files, the fate of which was established in the meristematic zone (Rost, 2011).

## Auxin, Cytokinin and Brassinosteroid Pathways Define the Transition Zone

The growth of a root is a measure of its ability to seek out and obtain water and nutrients from the soil. Three determinants of root growth are (1) the rate at which cells can be produced, (2) the point at which these cells transition from division to elongation, and (3) the growth potential of the cells. To understand the mechanisms that drive or constrain root growth under different environmental conditions it is necessary to identify the signaling pathways that regulate each of these three determinants. In roots, every major hormone group identified in plants has been shown to affect growth (Benková and Hejátko, 2009). In this review, we focus on auxins, cytokinins, and BRs, and discuss their collective roles in determining the transition point between cell division and elongation.

## The Antagonistic Role of Auxin and Cytokinins in Controlling Root Development

Auxin-dependent processes, including cell division and cell expansion, rely on auxin concentration gradients (Rahman et al., 2007; Perrot-Rechenmann, 2010; Wang and Ruan, 2013). In the RAM, a local reduction in active auxin (auxin minimum) coincides with the transition zone (Figure 1B) and is thought to be a key regulator of the switch from division to elongation/differentiation (Di Mambro et al., 2017). Although local biosynthesis occurs, auxin gradients found in the root system (Figure 1B) mainly result from auxin transport and degradation mechanisms (Kolomeisky, 2011; Di Mambro et al., 2017).

Directional auxin transport is mediated by PIN-FORMED (PIN) efflux carriers and other transmembrane proteins (Blilou et al., 2005). PINs are specifically expressed and polarly distributed within different root tissues. For example, PIN1 is found at the lower (rootward) flanks of cells in the central stele to direct auxin toward the root apex while PIN2 is consistently at the upper flanks of epidermal and lateral root cap cells. In the cortex, however, PIN2 occupies the lower flanks in the division zone, then flips to the upper flanks as cells enter the elongation zone (Feraru and Friml, 2008). Thus, PIN2 drives auxin flow away from the root apex once division ceases, suggesting that it plays a pivotal role in establishing the auxin minimum at the transition zone. The specific mechanisms orchestrating PIN polarity are the subject of ongoing research (Marhava, 2021) but endocytosis-mediated recycling plays a key role in modulating PIN activity.

Auxin-mediated cell proliferation is antagonized by cytokinins (Müller and Sheen, 2008), which promote cell differentiation in the transition zone of the root. In the transition zone, cytokinin activates the transcription factor ARABIDOPSIS RESPONSE REGULATOR1 (ARR1), which in turn activates GRETCHEN HAGEN 3.17 (GH3.17) and SHORT HYPOCOTYL 2 (SHY2). GH3.17 acts to conjugate free auxin with amino acids, which promotes auxin’s inactivation and degradation (Pierdonati et al., 2019). SHY2 represses the transcript levels of *PIN1*, *PIN2*, *PIN3* and *PIN7*, thereby preventing further shootward (PIN2) and rootward (PIN1, PIN3, PIN7) auxin transport (Dello Ioio et al., 2008; Di Mambro et al., 2017). Collectively, GH3.17 and SHY2 reduce the amount of auxin in the transition zone, which is associated with the developmental switch to elongation.

## Brassinosteroids Modulate Both Cell Division and Cell Elongation in the Root Meristem

The auxin gradient (Figure 1B), which trends from a maximum in the quiescent center and slowly diminishes toward the elongation zone (Petersson et al., 2009), is met by an opposing gradient of BR activity (Chaiwanon and Wang, 2015) and biosynthesis (Vukašinović et al., 2021; Figure 1C). In contrast to auxins, BRs act in close proximity to their synthesis, typically on cells in adjacent tissues or cell layers populated with BR receptors (Vukašinović and Russinova, 2018). BR-biosynthetic gene expression appears to be restricted to the inner tissues (Brady et al., 2007), and recent analysis indicates that enriched BR biosynthesis in the elongation zone is tightly orchestrated through the intercellular transport of some BR precursor molecules (Vukašinović et al., 2021). Translatome profiling indicates that BR-targeted gene activity is enriched in root epidermal cells (Vragović et al., 2015), consistent with evidence that confining BR signaling to the epidermis is sufficient for controlling meristem size (Hacham et al., 2011).

Balancing BR signaling is important for sustaining cell division in the RAM. BR signaling is initiated with the BR receptor, BRASSINOSTEROID INSENSITIVE 1 (BRI1), which is a plasma membrane-localized leucine-rich repeat receptor-like kinase (Belkhadir and Chory, 2006). BR binding to BRI1’s extracellular domain leads to the phosphorylation and activation of BRI1 SUPPRESSOR 1 (BSU1), a phosphatase that inactivates the BRASSINOSTEROID INSENSITIVE 2 (BIN2) glycogen synthase kinase 3 (Li and Nam, 2002; Mora-García et al., 2004; Kim et al., 2011). Degrading BIN2 removes the negative regulation of two transcription factors, BRASSINOZOLE-RESISTANT 1 (BZR1) and BRI1-EMS-SUPPRESSOR 1 (BES1), which are then targeted to the nucleus (Ryu et al., 2007; Tang et al., 2011) where they activate or repress target genes. BR application causes premature exit of cells from the division zone, leading to smaller meristems (González-García et al., 2011; Chaiwanon and Wang, 2015), while mutants defective in synthesizing or perceiving BR also have reduced meristem size (Hacham et al., 2011; Sakaguchi and Watanabe, 2017). In mutants with enhanced BR signaling, González-García et al. (2011) found that differentiated root epidermal cells were longer than those in the wild type and there was a reduced number of cells in the division zone.

## Negative Feedback: Cytoplasmic Linker Protein-Associating Protein Is Downregulated by Brassinosteroid but Also Sustains Brassinosteroid Signaling

**FIGURE 2:**
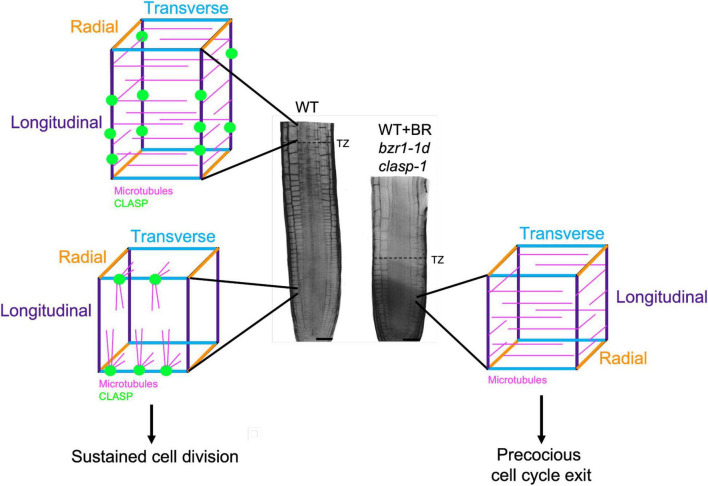
CLASP localization and microtubule organization in interphase cells in the root meristem. In the division zone of wild-type (WT) RAMs (lower left), CLASP (green) distributes to the sharp transverse edges (blue) formed from recent cell divisions. This promotes the formation of TFBs, allowing microtubules (pink) to grow around these edges. In the elongation zone of WT roots (upper left), CLASP localizes to the longitudinal edges (purple) and a primarily transverse array of microtubules forms respective to the axis of growth. In the division zone of plants with *CLASP* downregulated (*WT* + *BR, bzr1-1d*) or absent (*clasp-1*) (right), the lack of CLASP causes microtubules to adopt a primarily transverse array, which is associated with precocious cell cycle exit. This leads to a smaller meristem as marked by an earlier transition zone (dotted line on root) when compared to the WT.

A major breakthrough in understanding how BR inhibits cell production in the RAM was the identification of the microtubule-associated protein CLASP as one of its targets (Ruan et al., 2018). CLASP transcript-null mutants (*clasp-1*) (Ambrose et al., 2007; Ambrose et al., 2011) and mutants with enhanced BR signaling (González-García et al., 2011) display premature exit of cells from the RAM division zone (Figure 2). Based on this, Ruan and Wasteneys (2014) hypothesized that *CLASP* was regulated by the BR signaling pathway. It was subsequently confirmed that BR downregulates *CLASP* expression through the direct binding of the BZR1 and BES1 transcription factors to the *CLASP* promoter, and that BR treatments deplete CLASP protein levels with profound consequences on microtubule organization (Ruan et al., 2018). These observations indicate that BR signaling works predominantly through CLASP to regulate RAM size.

Intriguingly, CLASP also promotes the recycling of BRI1 receptors to the plasma membrane (Ruan et al., 2018) by stabilizing SNX1 endosomes along cortical microtubules (Ambrose et al., 2013). The direct interaction of CLASP with SNX1 thus completes a negative feedback loop. Accordingly, BR downregulation of *CLASP* expression will lead to a reduction in the number of BRI1 receptors on the plasma membrane, and dampened BR signaling. Conversely, increasing levels of CLASP will enhance BR signaling, eventually downregulating *CLASP* expression, which is likely to impact microtubule organization (discussed below in the section on Interphase Microtubule Arrays).

## Cytoplasmic Linker Protein-Associating Protein Fosters Pin-Formed 2 Recycling

How does the connection between CLASP and BR signaling impinge on the activity of the cytokinin-auxin signaling module in RAM development? In addition to promoting the recycling of BRI1, CLASP also promotes the recycling of PIN2 *via* its association with SNX1 endosomal compartments (Ambrose et al., 2013). In mutants that do not express *CLASP*, PIN2 levels are dramatically reduced at the plasma membrane, resulting in a pooling of auxin at the root tip (Ambrose et al., 2013). Given that BR treatments downregulate CLASP, we can postulate that PIN2 levels will also be reduced and auxin flow will be impaired. One objective for future investigations is to determine the extent to which meristem activity is dependent on CLASP and BR signaling operating upstream of auxin transport.

While we have focused here on just three hormones, the CLASP-BR feedback system likely impinges on other plant hormones. A recent study revealed a parallel pathway involving gibberellin (GA)-mediated localization of PIN2; high levels of GA were shown to promote PIN2 recycling to the plasma membrane, which required SNX1, CLASP, and an intact microtubule cytoskeleton (Salanenka et al., 2018). DELLA proteins (which are degraded by active GA signaling) interact with BZR1 to reduce growth (Li et al., 2012), so it would be interesting to determine how a combination of BR and GA would affect the CLASP-SNX1 sorting pathway in roots.

## Brassinosteroid and Cytoplasmic Linker Protein-Associating Protein-Dependent Changes in Interphase Microtubule Arrays in the Transition From Division to Elongation in the Root Apical Meristem

While CLASP has a critical role in mediating brassinosteroid and other hormone activity through the association of SNX1-associated endosomal compartments with cortical microtubules, we need to remember that CLASP is also a central player in microtubule dynamics and organization. Therefore, a key question is whether the downregulation of *CLASP* by the BR signaling pathway is central to the reorganization of microtubules as cells transition from proliferation to elongation.

As major drivers of cell division and directional expansion, microtubules display distinct changes in array organization as cells progress from division to differentiation. In defiance of the textbook paradigm that interphase cortical microtubule arrays are perpendicular to the major growth axis, it was demonstrated in 2000 that interphase microtubule in the *Arabidopsis* division zone in fact showed much greater angular deviation than in the elongation zone (Sugimoto et al., 2000). This observation was eventually explained by the identification of transfacial microtubule bundles (TFBs), prominent microtubule bundles that form at the newly formed transverse cell edges of interphase epidermal cells (Ambrose et al., 2011). TFBs are not observed once cells enter the transition zone (Ambrose et al., 2011), and with the onset of rapid elongation microtubules become predominantly transverse with respect to the axis of growth, which persists until growth rates begin to decline (Sugimoto et al., 2000).

The exact function of TFBs in maintaining the RAM has not been established, yet their presence is dependent on the activity of CLASP, and is strongly coupled with sustained cell proliferation. The precocious exit of cells from the division zone in *clasp-1* mutants is strongly coupled with changes in microtubule arrangements in epidermal cells (Ambrose et al., 2011). In the absence of CLASP, microtubules self-organize according to the geometrical constraints of cells. Specifically, microtubules encountering the sharper newly formed transverse cell edges of epidermal cells undergo catastrophe and TFBs fail to form whereas CLASP’s presence in wild-type cells fosters TFB formation (Ambrose et al., 2011). The absence of TFBs in *clasp-1* mutants results in a predominantly transverse microtubule orientation that resembles that found in the elongation zone (Ambrose et al., 2011). This finding, along with other studies, underscore the importance of identifying the regulatory mechanisms controlling the activity of MAPs such as CLASP and END BINDING PROTEIN 1 (EB1) (Ambrose et al., 2011; Molines et al., 2018).

In addressing the question of whether the downregulation of CLASP by the BR signaling pathway is central to the reorganization of microtubules in the transition from proliferation to elongation, Ruan et al. (2018) demonstrated that BR treatments or enhanced BR signaling with the constitutively active *bzr1-1D* mutant reduce CLASP levels and cause failure of TFBs to form, resulting in transverse cortical microtubule arrays in the division zone epidermal cells and early transition to elongation (Figure 2). While the mechanism behind the spatiotemporal regulation of CLASP is currently under investigation, there are two possible ways in which this could occur. First, in the transition and elongation zones, where BR signaling is high (Chaiwanon and Wang, 2015), a marked reduction in *CLASP* expression could prevent the formation of TFBs. This is consistent with the transverse microtubule arrays observed in elongating cells that have exited the division zone. Second, CLASP levels may build up then gradually drop off over the course of each cell cycle. This idea is related to the observation that TFBs are found in interphase cells and disappear when the preprophase band forms and the cell prepares for division. While this is a tempting model, additional research is needed to determine if there is a connection between BRI1 and CLASP oscillations throughout a single cell cycle.

CLASP’s function in the formation of TFBs relates to its role as a rescue factor in enabling the growth of microtubules around sharp cell edges (Ambrose et al., 2011). It remains possible, however, that CLASP works closely with the microtubule-bundling proteins MAP65-1 and MAP65-2, which are expressed and active in the RAM (Lucas and Shaw, 2012). *In vitro* experiments have indicated that MAP65-1 and MAP65-2 increase the flexibility of both independent and bundled microtubules, suggesting that they could mediate steep contact angle bundling (Portran et al., 2013), which occurs with TFBs. It is compelling to note that in animal and yeast cells, MAP65 orthologs directly interact with and target CLASP to regions of microtubule overlap such as the mitotic spindle (Bratman and Chang, 2007; Liu et al., 2009). Once directed to the spindle, CLASP stabilizes the spindle, which in the case of mammalian CLASP1 has been shown to allow for proper chromosomal segregation (Liu et al., 2009). In Arabidopsis, specifically, it has been suggested that similarities in the *map65-1 map65-2* and *clasp-1* mutant hypocotyl phenotypes point to a potential role for MAP65-1 and MAP65-2 in localizing CLASP to cortical bundle sites (Lucas et al., 2011). This relationship remains an open area of investigation and could underlie the formation of TFBs in Arabidopsis and the indispensable role they play in the promotion of meristematic proliferation.

## Root Meristem Growth Is Light- and Sucrose-Dependent

Roots are highly responsive to mobile signals derived from the shoot, including photosynthetic sucrose, hormones such as abscisic acid (ABA), and the transcription factor HY5 (Kircher and Schopfer, 2012; Chen et al., 2016; Ha et al., 2018). In the absence of light, roots do not receive these signals and rapid hypocotyl elongation is prioritized over cotyledon and root development until favorable conditions are achieved.

**FIGURE 3:**
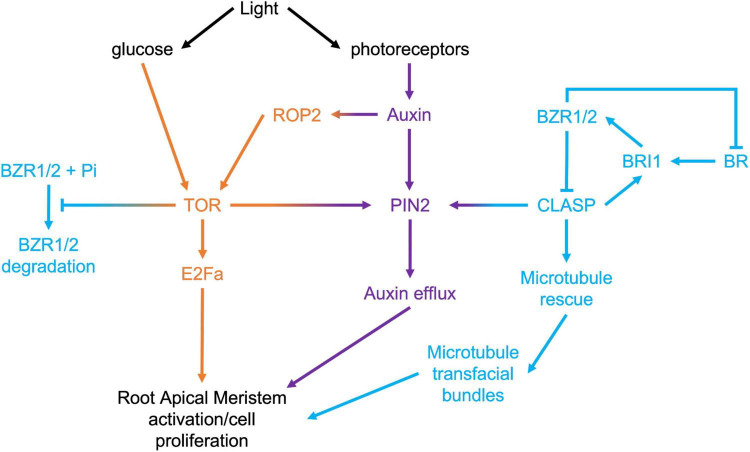
Signaling pathways that converge in the regulation of root apical meristem growth.

Target of Rapamycin (TOR) is a highly conserved eukaryotic protein kinase that integrates environmental signals with downstream developmental and metabolic pathways, such as translation, protein degradation, and cell division (Dobrenel et al., 2016). In plants, TOR is activated by light through two pathways (Figure 3): phytochrome-mediated and sugar-mediated (Xiong et al., 2013; Chen et al., 2018). Within the context of exploring the mechanisms driving meristem activity, it is intriguing that TOR activity is pivotal in both brassinosteroid signaling and PIN2-mediated auxin transport. Under prolonged darkness, autophagy degrades the BR transcription factor BZR1, leading to collapse of the BR pathway (Zhang et al., 2016). Consistent with TOR’s inhibition of autophagy (Liu and Bassham, 2010), activating TOR with exogenous sugar prevents the degradation of BZR1, and promotes hypocotyl cell elongation (Zhang et al., 2016). It has also been shown that TOR can directly phosphorylate and stabilize PIN2 and that this plays a key role in maintaining the auxin gradient within the RAM (Yuan et al., 2020).

Given that two of the key mechanisms under direct regulation by TOR, specifically PIN2-mediated auxin transport, and BRI1-mediated BR signaling, are sustained by CLASP, an important avenue for exploration will be to determine the relationship between TOR and CLASP in RAMs. Recent evidence has confirmed that CLASP activity in roots is tightly controlled by light availability. Halat et al. (2020) used dark-grown root meristems to study how *CLASP* levels were regulated when rapid root growth was not required. In the absence of light, they found that CLASP translation was blocked, leading to a collapse of cell proliferation. Somewhat paradoxically, but consistent with the shut-down of RAM-specific BR signaling in the absence of light and the translational block, *CLASP* transcripts were greatly elevated under these conditions. Miotto et al. (2019) analyzed the transcriptional profile of roots when only the shoot was exposed to light and compared this to plants grown in total darkness. They found that *CLASP* was downregulated by light 7 days after germination, which supports the Halat et al. (2020) finding of elevated *CLASP* transcript in dark conditions with reduced translation. CLASP protein levels were highly reduced on cell edges in the dark, and consequently, microtubule organization and the abundance of BRI1 receptors and PIN2 auxin transporters at the plasma membrane were negatively impacted.

The Halat et al. (2020) study determined that adding sucrose to the growth media of dark-grown wild-type or *clasp-1* plants could only partially restore root elongation, and that this treatment had no significant effect on cell division rates, and instead only stimulated cell elongation. While supplementing dark-grown seedlings with sucrose is likely to stabilize PIN2 *via* TOR kinase phosphorylation (Yuan et al., 2020), evidently this is insufficient to stimulate cell division. Importantly, sucrose also stimulated cell elongation but no division in *clasp-1* mutants, indicating that CLASP is tightly connected to light-mediated pathways that stimulate cell division in the RAM (Halat et al., 2020). These findings suggest that instead of promoting root growth in an environment where hypocotyl elongation is critical for survival, plants strategically allocate resources and only activate root cell division when the seedling has a sustainable source of light. Moving forward, researchers should carefully consider whether the addition of sucrose affects their phenotype of interest.

## Outstanding Questions, Technical Challenges, and Advances

### Cellular Geometry, Tensile Forces, and Microtubule Organization

Understanding the factors that influence developmental transitions is crucial for studying RAM growth, but many of the underlying mechanisms remain elusive. An area of study that has emerged since Ambrose et al. (2011) first modeled microtubule organization in 3-dimensions is the cytoskeletal response to changes in cellular geometry. In the *Arabidopsis* root, most cells are hexagonal but range in shape from flattened to isodiametric cuboids in the division zone to highly elongated prisms and or hemicylinders in the elongation zone. The balance between the forces exerted by internal turgor pressure within the cell and the counteracting rigidity of the cell wall determine the directionality of cell growth. How are internal and external mechanical forces transduced to the microtubule cytoskeleton? Microtubule orientation patterns can be mediated by changing the force on the substratum in *in vitro* gliding motility assays (Inoue et al., 2015) and microtubules have been shown to polymerize at faster rates under higher tensile forces (Trushko et al., 2013). One theory based on *in vivo* data posits that cortical microtubules spontaneously align along the direction of maximal tensile stress in plant cells, which drives the ordered deposition of cellulose microfibrils (Hamant et al., 2008). In rapidly elongating cells, tensile stress is highest in the circumferential direction along which microtubules align to reinforce the cell wall so that the cell continues to grow anisotropically (Trinh et al., 2021). As tensile forces change, microtubules are clearly responsive to these mechanical cues, but it is still unclear whether this is an autonomous process or whether microtubule organization is dictated by certain features of cell geometry, such as sharp cell edges (Ambrose et al., 2011). Cell edges were historically considered unique microdomains of the cell for cytoskeletal organization. In the water fern *Azolla*, microtubules were observed in clusters at edges in post-cytokinetic cells (Gunning, 1980). Studies in *Arabidopsis* have built upon these observations by showing that GAMMA TUBULIN COMPLEX PROTEINS2 and 3 (GCP2 and GCP3) are enriched at cell edges in the root (Ambrose and Wasteneys, 2011). This lends support to the theory that cell edges can act as a site for microtubule nucleation. Computational modeling of plant cell shapes has been an informative tool that can recapitulate the observed microtubule patterns *in vivo* by inputting parameters such as catastrophe events induced by cell edge curvature (Ambrose et al., 2011; Chakrabortty et al., 2018). Recent experimental evidence suggests that microtubules also reorient by mechanisms that do not rely on cell edges. By removing the cell walls and generating protoplasts, Colin et al. (2020) found that microtubules conform to cell geometry by default, then switch their alignment to match the direction of tensile stress when internal turgor pressure increases.

### A Dark-Root System

Several technical challenges for studying RAM growth are currently being addressed in the field to obtain more accurate data on the transition from division to differentiation. One limitation of the current studies on root growth is that plants are either completely exposed to light or grown in total darkness. In a natural setting, the aerial tissues of a plant are exposed to light, while roots grow beneath the soil in near or complete darkness. The recently developed D-Root system is a device that allows one to grow plants with the shoots exposed to light, while the root system remains in the dark. Under these conditions, roots grew longer and were more resistant to salt and osmotic stress (Silva-Navas et al., 2015). These types of experimental setups will be an interesting way to test how proteins and hormone signals in the root respond to the natural circadian signals that plants receive on a daily basis.

### Long-Term and Deep Tissue Imaging

One of the most significant roadblocks to time-lapse imaging is bleaching of fluorophores after prolonged laser exposure. To improve the imaging of MAPs and microtubules throughout the cell cycle, more sophisticated microscopy technologies (such as lattice light sheet technology) should be employed. These platforms will produce greater resolution of subcellular structures to enable more accurate quantifications of changes in these proteins over time and space. Advanced imaging modalities such as lattice light sheet microscopy allow users to image deep into the root, which is critical since the tissue of interest may be covered by the lateral root cap or other tissue layers. Technology that permits simultaneous two-color imaging is especially important for observing dynamic changes in microtubules (that often grow at speeds of ∼ 7 μm/min) and MAPs in the different developmental stages of the root.

### Hormone Sensors

The emerging importance of phytohormone gradients in defining transition zones in plant development places increasing demand for biosensors that can quantify changes in hormone levels on a cellular scale in different tissue types of roots. Isoda et al. (2021) provide a comprehensive review of available phytohormone sensors; here we briefly highlight how some recent advances for the detection of auxins, BRs, cytokinins, ABA and GAs in the RAM.

Until recently, auxin levels have been inferred indirectly from probes that generate outputs related to auxin-mediated signaling. The DR5 reporter and its more-sensitive follow up, DR5v2, exploit auxin response elements to drive the transcription and synthesis of green fluorescent protein or other reporters (Ulmasov et al., 1997; Liao et al., 2015). By contrast, the nuclear localized DII-Venus reporter is a fusion of the degradation domain of the AUX/IAA repressor with the Venus fluorescent protein (Brunoud et al., 2012). By measuring auxin activity through AUX/IAA degradation-mediated loss of fluorescence rather than downstream transcriptional activity, DII-Venus, and its dual-fluorescent ratiometric derivative, R2D2 (Liao et al., 2015) detect early auxin signaling and thus greatly improve temporal fidelity. Unfortunately, neither of these systems directly quantifies auxin levels. Recently, Herud-Sikimić et al. (2021) engineered a fluorescent auxin biosensor that works by driving fluorescence resonance energy transfer in response to auxin binding. This new development promises to re-map the spatio-termporal dynamics of auxin. In contrast to auxins, monitoring BRs has been limited to quantifying BR activity through the distribution of the fluorescently tagged BR-activated transcription factor BZR1, the intensity of which increases as cells enter the elongation zone (Chaiwanon and Wang, 2015). Matrix-assisted laser desorption/ionization time-of-flight mass spectrometry has been employed to measure cytokinins and ABA levels in the roots of rice (Shiono et al., 2017). This study revealed contrasting longitudinal gradients, with the cytokinin trans-zeatin having the strongest signal 40 mm away from the root tip in rice, while ABA was concentrated nearest the root tip (Shiono et al., 2017). Finally, a FRET-based detector called GPS1 revealed that GAs are at low levels in the division zone and peak in the elongation zone (Rizza et al., 2017). Together, these studies indicate that auxin and ABA have highest levels in the division zone, whereas BRs, cytokinins, and GAs are more abundant in elongating cells.

### Computer Modeling

The roles of hormone pathways in growth and development have been elucidated to date by mutating pathway components and observing resultant phenotypes. The prevalence of crosstalk between different hormone pathways presents great challenges to attributing specific effects to these perturbations. This has pushed the development of computer models, allowing researchers to manipulate desired variables and rapidly observe the potential outcomes prior to proceeding with time-consuming bench work. One recent example is a computer model for auxin-dependent root growth developed by Marconi et al. (2021) that combines the characteristics of polar auxin transport with an anisotropy factor that accounts for the effect of cell wall components and cytoskeletal elements. Considering the root transition zone specifically, this model successfully replicated known changes in PIN subcellular distribution, prompting the authors to suggest that this arises from conflicting shootward and rootward flows of auxin in the external and internal tissues, respectively, and to propose that this polarity flip could drive auxin diffusion from the outer tissues into the inner tissues to promote continual root growth. This is an important finding as it identifies a role for polar auxin transport, cell wall components, and cytoskeletal elements in establishing a key transition point within the root. To test the strength of their model, Marconi et al. (2021) simulated PIN knockdowns and chemical disruption of cortical microtubules. They were able to successfully generate findings consistent with previous *in vivo* experiments providing support for the use of their model in future investigations into auxin-dependent root growth. Computer modeling that can recapitulate plant growth patterns will likely be an important tool to guide *in vivo* experimentation.

## Discussion

The growth and development of plant meristems depends on careful coordination between cell proliferation and differentiation. Within the field of root meristem research, growth has been examined in the context of chemical messengers, the cytoskeleton, and input from environmental signals. Improvements in imaging technologies have already provided unprecedented information on how plants modulate their growth at the subcellular level across multiple tissue layers in real time. Recent work has highlighted a crucial role for CLASP in a negative feedback-based regulatory mechanism of root meristem development that involves auxin, cytokinin, and BR. It is conceivable that the epidermis is the “master regulator” of RAM growth, and CLASP plays a major role in this tissue by integrating hormone signaling with microtubule organization. Bearing in mind that there are BRI1-LIKE receptors in the inner root tissues, it would be interesting to investigate if CLASP is involved in a similar pathway in these cell types or if its functional relationship with SNX1 is confined to the epidermis.

## Author Contributions

All authors contributed to the writing of this manuscript.

## Conflict of Interest

The authors declare that the research was conducted in the absence of any commercial or financial relationships that could be construed as a potential conflict of interest.

## Publisher’s Note

All claims expressed in this article are solely those of the authors and do not necessarily represent those of their affiliated organizations, or those of the publisher, the editors and the reviewers. Any product that may be evaluated in this article, or claim that may be made by its manufacturer, is not guaranteed or endorsed by the publisher.
